# Comparison of two different techniques for balloon sizing in percutaneous mitral balloon valvuloplasty: which is preferable?

**DOI:** 10.5830/CVJA-2015-062

**Published:** 2016

**Authors:** Ahmet Tastan, Ali Ozturk, Omer Senarslan, Erdem Ozel, Samet Uyar, Emin Evren Ozcan, Omer Kozan

**Affiliations:** Department of Cardiology, Sifa University Faculty of Medicine, Izmir, Turkey; Department of Cardiology, Sifa University Faculty of Medicine, Izmir, Turkey; Department of Cardiology, Sifa University Faculty of Medicine, Izmir, Turkey; Department of Cardiology, Sifa University Faculty of Medicine, Izmir, Turkey; Department of Cardiology, Sifa University Faculty of Medicine, Izmir, Turkey; Department of Cardiology, Sifa University Faculty of Medicine, Izmir, Turkey; Department of Cardiology, Dokuz Eylul University Faculty of Medicine, Izmir, Turkey

**Keywords:** mitral balloon valvuloplasty, balloon size, echocardiography

## Abstract

**Background:**

Percutaneous balloon mitral valvuloplasty (BMV) is an important option for the treatment of mitral valve stenosis. The crux of this process is choosing the appropriate Inoue balloon size. There are two methods to do this. One is an empirical formula based on the patient’s height, and other is to choose according to the maximal inter-commissural distance of the mitral valve provided by echocardiography.

**Methods:**

The study, performed between January 2006 and December 2011, included 128 patients who had moderate to severe mitral stenosis and whose valve morphology was suitable for BMV. Patients were randomised into two groups. One group was allocated to conventional height-based balloon reference sizing (the HBRS group) and the other was allocated to balloons sized by the echocardiographic measurement of the diastolic inter-commissural diameter (the EBRS group).

**Results:**

BMV was assessed as successful in 60 (92.3%) patients in the HBRS group and in 61 (96.8%) in the EBRS group (*p* = 0.03). The mean of the calculated balloon reference sizes was significantly higher in the HBRS than in the EBRS group [26.3 ± 1.2 mm, 95% confidence interval (CI): 26.1–26.6 vs 25.2 ± 1.1, 95% CI: 25.0–25.4, respectively; *p* = 0.007). Final mitral valve areas (MVA) were larger and mitral regurgitation (MR) > 2+ was less in the EBRS group (*p* = 0.02 and *p* = 0.05, respectively).

**Conclusions:**

EBRS is a method that is independent of body structure. Choosing Inoue balloon size by measuring maximal diastolic annulus diameter by echocardiography for BMV may be an acceptable method for appropriate final MVA and to avoid risk of significant MR.

## Background

Although both the incidence and prevalence of rheumatic heart disease have seen a dramatic decrease in recent years, it is still the leading cause of mitral valve stenosis. It is known that rheumatic changes are present in 99% of stenotic mitral valves excised at the time of mitral valve surgery.^1^

Mitral valve stenosis is the most serious sequel of rheumatic fever and it has unfavourable effects on survival rates and quality of life. The disease is endemic in developing countries, including Middle Eastern countries.^2^ Long-term left heart obstruction causes unwanted structural and haemodynamic deterioration. Medical therapy neither treats the disease nor modifies its course.^1^ In addition medical therapy is routinely given to reduce symptoms and to prevent thromboembolic complications, or to avoid recurrent rheumatic fever.

Since the first publication of the Inoue balloon in 1984, balloon mitral valvuloplasty (BMV) has become the procedure of choice all over the world because of its lower cost and morbidity rate.^3^ Successfully applied BMV improves the haemodynamics and symptoms related to mitral valve stenosis, and has favourable impacts on early and long-term survival of patients.^4^,^5^

According to recently published valvular heart disease guidelines, BMV is recommended for symptomatic patients with moderate to severe mitral valve stenosis. Candidate patients for BMV should have a suitable valve structure with no mitral regurgitation (MR). BMV should not be administered to a patient with thrombus in the cardiac chambers. The main purpose of BMV is to provide an adequate mitral valve area (MVA) of > 1.5 cm^2^ with no significant MR (MR not more than 2/4 in over 80% of patients).^6^ Despite high technical expertise in BMV, MR remains a major procedure-related complication.^7^

Many studies have shown that acute procedural results, including final MVA and post-procedural MR, independently predict the long-term outcome after BMV.^8^ The incidence of severe MR after BMV reported in the literature varies between 1.4 and 7.5%.^9^,^10^ BMV is recommended for symptomatic patients with suitable valvular morphology who have moderate to severe mitral stenosis (MVA < 1.5 cm^2^) and also for asymptomatic patients with pulmonary artery systolic pressure of > 50 mmHg at rest or > 60 mmHg with exercise.11

The aim of this procedure is to provide adequate valve area while protecting the mitral valve apparatus. For this purpose, selection of the appropriate balloon size is one of the most critical factors for BMV.^12^ There are two basic methods to decide the size of the Inoue balloon. One is the use of an empirical formula to calculate inflated balloon catheter diameter based on the height of the patient [size = 0.1 × height (cm) + 10].^13^,^14^ Differences between body habitus, heart orientation and configuration of the cardiac skeleton are combined to calculate balloon diameter using a height-based formula. Therefore, different results can be obtained, even in patients with the same height.

A reasonable alternative approach is choosing balloon size according to the maximal inter-commissural distance of the mitral valve as provided by echocardiography. This is a more direct and conservative method of balloon sizing to avoid possible mistakes when determining the size of the balloon, and to prevent possible complications in the procedure.

The aim of this study was to assess the early haemodynamic and echocardiograhic results of BMV according to these two different types of Inoue balloon selection strategy.

## Methods

Between 1 January 2006 and 31 December 2011, 128 consecutive symptomatic patients were seen with moderate to severe mitral stenosis (MVA < 1.5 cm^2^), whose valve morphology was suitable for BMV and who had no contraindications for BMV. Symptomatic and asymptomatic patients were included who had a pulmonary artery systolic pressure of > 50 mmHg at rest or 60 mmHg with exercise in the presence of favourable morphological criteria of the mitral valve, derived from the echocardiographic Wilkins scoring system.^11^ Patients were considered eligible if they were aged 18 years or older.

Exclusion criteria were mild mitral stenosis, a Wilkins score of > 10, moderate or severe mitral regurgitation, any moderate or severe valvular heart disease other than mitral stenosis, a history of coronary artery disease, heart failure, pulmonary embolism, congenital heart disease, chronic kidney disease and current pregnancy.

Patients were randomised into two groups. One group was allocated to conventional height-based balloon reference sizing (the HBRS group) and the other was allocated to balloons sized by the echocardiographic measurement of the diastolic intercommissural diameter (the EBRS group). BMV was performed in all patients.

All patients were informed about the study, and written consent was obtained. The study was approved by the institution’s ethics committee and performed in accordance with the Helsinki Declaration.

## Echocardiography

In accordance with the recommendations of the American Society of Echocardiography, all transthoracic echocardiographic (TTE) examinations were performed with the patient lying in the left lateral decubitus position, and two-dimensional images were recorded and measured in the apical four-chamber, two-chamber, and parasternal long- and short-axis views.^15^ Echocardiographic examinations were performed by two independent cardiology specialists, blinded to the outcome of BMV, in the echocardiography laboratory before and one month after BMV.

B-mode, two-dimensional Doppler and colour-flow Doppler echocardiographic evaluations were performed using a Siemens Acuson CV70 system (Siemens AG Medical Solutions, Erlangen, Germany) with a 2.5–4.0-MHz transducer with second harmonic capabilities. Transoesophageal echocardiographic (TEE) examinations were performed on all patients with a 5-MHz multiplane endoscopic probe.

MVAs were calculated using the direct planimetry and pressure half-time method. Diastolic trans-mitral gradients were measured by continuous-wave Doppler echocardiography. Systolic pulmonary artery pressure (sPAP) was measured with continuous-wave Doppler. Tricuspid regurgitation velocity (V) was recorded from any view and used to determine sPAP (sPAP = 4V^2^ + right atrial pressure). V is the maximum velocity of the tricuspid valve regurgitant jet, measured by continuous-wave Doppler, added to the estimated right atrial pressure. Right atrial pressure was calculated using the caval respiratory index, as described by Kircher *et al*.^16^

Finally, the maximal inter-commissural distance was measured on the parasternal short-axis view from the anterolateral to posteromedial commissures in mid-diastole. The degree of mitral regurgitation and the presence of concomitant valvular heart disease and left atrial clot were also determined.

## Catheterisation and valvuloplasty

Cardiac catheterisation was performed using Siemens AXIOM Artis dFC equipment (Siemens AG, Medical Solutions, Erlangen Germany). The right and left heart haemodynamic study was performed to evaluate sPAP and mitral valve pressure gradients.

In the HBRS group, balloons were selected using the height-based reference size by calculating according to the standard height-based formula [0.1 × height (cm) + 10]. In the EBRS group, balloons were selected according to the echocardiographic inter-commissural distance measurement in mid-diastole.

The classic antegrade Inoue balloon technique was used for BMV by two experienced cardiologists in a standard and similar fashion. Procedures were performed with TEE guidance. After the atrial septostomy and appropriate septal dilation, 100 IU/kg of heparin was administered to achieve an activating clotting time of > 250 seconds. Left ventriculography was performed before and after BMV to evaluate mitral regurgitation. A successful BMV was defined as an uncomplicated procedure yielding a final mitral valve area of > 1.5 cm2 and post-valvuloplasty mitral regurgitation of < 3+.

## Statistical analysis

Continuous variables are given as mean ± SD. Categorical variables were defined as percentages and compared with the chi-square test to compare the measurements before and after BMV, and a Student’s paired t‑test was used. A probability value of p *p* 0.05 was considered significant, and two-tailed *p*-values were used for all statistics.

## Results

In this study, 128 patients participated. In 65 patients, Inoue balloon sizes were calculated according to HBRS, while in the remaining 63 patients, sizes were provided by EBRS. The study patients’ baseline clinical, echocardiographic and catheterisation characteristics are shown in Tables 1,2 and 3.

**Table 1:** Background characteristics of study patients

	*HBRS (n = 65)*	*EBRS (n = 63)*	*p-value*
Age (years)	35.4 ± 6.2	31.4 ± 5.1	0.12
Gender, n (%)			
Male	18 (27.7)	17 (27)	0.67
Female	47 (72.3)	46 (73)	0.58
Weight (kg)	66.2 ± 10.9	70.1 ± 11.1	0.14
Height (cm)	162 ± 9	163 ± 9	0.62
CVD	3	1	0.09
Atrial fibrillation	14	12	0.53
NYHA			
1	16	12	0.62
2	41	42	
3	8		9

EBRS, echocardiographic balloon reference sizing; HBRS, height-based balloon reference sizing; F, female; M, male; CVD, cerebrovascular disease; NYHA, New York Heart Association functional classification.

**Table 2:** Pre-procedural echocardiographic data of study participants

	*HBRS (n = 65)*	*EBRS (n = 63)*	*p-value*
LVDD (mm)	46 ± 2.4	47 ± 2.5	0.81
LVSD (mm)	29 ± 1.5	29 ± 1.4	0.78
LA (mm)	49 ± 6	47 ± 6	0.51
LVEF (%)	64 ± 5	65 ± 5	0.54
MVA (cm^2^)	1.1 ± 0.2	1.0 ± 0.3	0.13
MV Wilkins score	8.7 ± 1.1	9.1 ± 1.4	0.38
MV max gradient (mmHg)	22.4 ± 5.5	21 ± 5.3	0.34
MV mean gradient (mmHg)	9.5 ± 4.2	9.7 ± 4.5	0.69
MR			
0	20	19	0.52
1+	42	39	
2+	3	5	

EBRS, echocardiographic balloon reference sizing; HBRS, height-based balloon reference sizing; LVDD, left ventricular end-diastolic diameter; LVSD, left ventricular end-systolic diameter; LA, left atrium; LV EF, left ventricular ejection fraction; MVA, mitral valve area; MV, mitral valve; MR, mitral regurgitation.

**Table 3:** Pre-procedural catheterisation data of study participants

**	*HBRS (n = 65)*	*EBRS (n = 63)*	*p-value*
PA pressure (mmHg)	49 ± 15	51 ± 16	0.91
RV pressure (mmHg)	48 ± 14	49 ± 15	0.82
RA pressure (mmHg)	7.5 ± 2	9.1 ± 2.4	0.14
MV mean pressure (mmHg)	9.9 ± 4.1	10.4 ± 4.4	0.32
MR			
0	21	20	0.69
1+	41	39	
2+	3	4	

EBRS, echocardiographic balloon reference sizing; HBRS, height-based balloon reference sizing; PA, pulmonary artery; RV, right ventricle; RA, right atrium; MV, mitral valve; MR, mitral regurgitation.

The mean age of the patients was 32 years in the HBRS group and 31 in the EBRS group, and there was no statistically significant difference (*p* = 0.16) between the groups; 72.7% of the patients were female. There was no difference between the groups in terms of weight and height (*p* = 0.64 and *p* = 0.62, respectively). Therefore it can be considered that both groups were similar in terms of body mass index.

Echocardiographic and catheterisation assessment of patients before the procedure showed no significant differences between the basic features of the two groups. The degree of mitral valve stenosis, mitral valve scores and MR (2+) were similar between the two groups (*p* = 0.73, *p* = 0.58 and *p* = 0.74, respectively).

BMVs were performed successfully on both groups. Adequate mitral valve area and a decrease in MVG were obtained.

BMV was assessed as successful in 60 (92.3%) patients in the HBRS group and in 61 (96.8%) in the EBRS group (*p* = 0.03). The mean of the calculated balloon reference sizes was significantly higher in the HBRS than in the EBRS group [26.3 ± 1.2 mm, 95% confidence interval (CI): 26.1–26.6 vs 25.2 ± 1.1, 95% CI: 25.0– 25.4, respectively; *p* = 0.007]. The final inflated balloon sizes were similar between the groups (25.6 ± 0.9 mm, 95% CI: 25.3–25.9 vs 25.9 mm ± 1.0 mm, 95% CI: 25.1–25.8, respectively; *p* = 0.34).

The post-procedural results of the patients are presented in Table 4. A greater decrease in the trans-mitral mean gradient was observed in the EBRS group but it was not statistically significant (*p* = 0.06). In the EBRS group, larger MVAs were achieved than in the HBRS group (1.6 ± 0.3 cm^2^, 95% CI: 1.56–1.69 vs 1.7 ± 0.3 cm^2^, 95% CI: 1.57–1.74, respectively; *p* = 0.02). The incidence of significant MR (3–4+) was lower than in the HBRS group (*p*p = 0.05 by echocardiography and p = 0.03 by ventriculography). Also, 2+ degree MR was significantly less developed in the EBRS patients (*p* = 0.01).

**Table 4:** Post-procedural catheterisation data of study participants

	*HBRS (n = 65)*	*EBRS (n = 63)*	*p-value*
Estimated reference MBS (mm)	26.3 ± 1.2	25.2 ± 1.1	0.02
Final balloon size	25.6 ± 0.9	25.9 ± 1.0	0.34
MVA (cm^2^)	1.6 ± 0.2	1.7 ± 0.3	0.02
Transmitral mean gradient (mm Hg)	3.2 ± 0.3	3.0 ± 0.3	0.04
PAP (mmHg)	28 ± 8	29 ± 7	0.81
MR severity, *n* (%)			
Echocardiography			
0	10	14	0.03
1+	41	43	
2+	11	5	
3-4+	3	1	
Catheterisation			
0	9	13	0.04
1+	39	42	
2+	13	6	
3-4+	4	2	

EBRS, echocardiographic balloon reference sizing; HBRS, height-based balloon reference sizing; MBS, mitral balloon size; MVA, mitral valve area; PAP, pulmonary artery pressure; MR, mitral regurgitation.

## Discussion

In recent years, Inoue balloon mitral commissurotomy has become the treatment of choice in many patients with rheumatic mitral stenosis. The main target of this procedure is to resolve the stenotic mitral orifice without causing extensive damage to the commissures, leaflets and subvalvular apparatus, thus leading to excessive mitral regurgitation. The most common serious complication is haemopericardium, with an incidence of 0 to 2.0%.^17^ Severe MR is another important and common serious complication after BMV.^18^

Many studies have shown that acute procedural results, including final MVA and post-procedural MR, independently predict the long-term outcome after BMV.^8^ When severe mitral regurgitation occurs after BMV, surgical treatment is required at some point. Most mild but significant cases of MR are caused by commissural split, chordal rupture or leaflet laceration.

Although there are many data for MBV, there is no consensus in determining the optimal size of the balloon.^19^ Appropriate balloon catheter sizing is the most important step for successful MBV procedure, as well as in reducing complications.^20^ Routine balloon sizing based on the conventional height-based formula has been validated in many studies.^21^ However, empirical selection of balloon size by the height-based formula has no correlation with variables such as cardiac structure, MVA and orifice. This mismatch can prevent the success of the process required and can lead to inappropriate consequences, even though perfect procedures are carried out by trained surgeons. Also, the relationship of a person’s height to the diameter of the mitral valve orifice is not necessarily linear.^12^ As a result of these findings, more effective methods have been investigated to determine the appropriate size of the balloon to maximise success and efficiency and to minimise complication rates.^22^

Nobuyoshi and colleagues recommended selecting the balloon size by directly measuring the mitral annular diameter using two-dimensional echocardiography to avoid undesirable extensive injury to the mitral valve apparatus.^12^ When maximal diastolic annulus diameter is used for balloon sizing, the balloon reference sizes are smaller than those obtained with the heightbased formula.^22^ In this way, balloons with a smaller diameter can be used to achieve sufficient mitral valve area, and with smaller balloons, procedures might be performed with less damage to the chordal structure and the leaflets. Less damage to the mitral valve apparatus results in less MR.

In our study we have shown that selecting Inoue balloon size according to echocardiographic maximal diastolic diameter is as efficient as using the height-based formula. Final balloon sizes were similar between the HBRS and EBRS groups but were smaller in the EBRS group. There was a significant difference between calculated and final balloon sizes in the HBRS group. We achieved sufficient MVAs by echocardiography-derived balloon sizing in this study, associated with lesser degrees of MR change.

Sanati and colleagues had the same clinical results with the EBRS method. Their final balloon sizes were similar between the two groups, and they achieved better valve areas with less MR.^22^ We believe that if calculated and final balloon sizes are similar, the success of MBV will be higher. Using the correct size of balloon and inflation pressure will cause less damage to the mitral valve apparatus.

Considering the fact that severe MR is also infrequent with the height-based method, but in our study severe MR was seen less in the EBRS group, we believe that applying invasive treatments for heart diseases using imaging methods could be better than using empirical methods. So in order to achieve effective MVA without severe MR, we suggest the EBRS method to select Inoue balloon size. In the future, the importance and correlation of MR developing after MBV with prognosis of patients will be better understood.

## Study limitations

The participants in this study were all suitable for MBV, and we did not include patients who had a borderline Wilkins score. The number of patients in our study was higher than in other studies in the literature; however, the number of patients should perhaps have been greater. Also, long-term follow-up results, especially those concerning MR, are yet to be published.

## Conclusion

EBRS is a method that is independent of body structure. Some patients are tall, some are short, and some are obese or asthenic, so patients who have discordance between heart size and body structure may benefit from this method. Choosing Inoue balloon size for BMV by measuring maximal diastolic annulus diameter using echocardiography is a reasonable method with acceptable final MVAs to avoid the risk of significant MR.

Echocardiographic balloon sizing for BMV should be used, especially in patients with discordance between height and heart size. We believe that all types of invasive procedures may be planned according to the dimensions of cardiac structures. Our study sheds some light on this issue.
